# Research Trends and Dynamics in Single-cell RNA Sequencing for Musculoskeletal Diseases: A Scientometric and Visualization Study

**DOI:** 10.7150/ijms.104697

**Published:** 2025-01-01

**Authors:** Siyang Cao, Yihao Wei, Yaohang Yue, Deli Wang, Ao Xiong, Jun Yang, Hui Zeng

**Affiliations:** 1 National & Local Joint Engineering Research Centre of Orthopaedic Biomaterials, Peking University Shenzhen Hospital, Shenzhen, Guangdong, People's Republic of China.; 2 Shenzhen Key Laboratory of Orthopaedic Diseases and Biomaterials Research, Peking University Shenzhen Hospital, Shenzhen, Guangdong, People's Republic of China.; 3 Department of Bone & Joint Surgery, Peking University Shenzhen Hospital, Shenzhen, Guangdong, People's Republic of China.; 4 Department of Rehabilitation Science, The Hong Kong Polytechnic University, Hong Kong Special Administrative Region, People's Republic of China.; 5 Faculty of Pharmaceutical Sciences, Shenzhen Institute of Advanced Technology, Chinese Academy of Sciences (CAS), Shenzhen, Guangdong, People's Republic of China.; 6 Faculty of Pharmaceutical Sciences, Shenzhen University of Advanced Technology, Shenzhen, Guangdong, People's Republic of China.; 7 Department of Radiology, Peking University Shenzhen Hospital, Shenzhen, Guangdong, People's Republic of China.; 8 Department of Orthopedics, Shenzhen Second People's Hospital, The First Affiliated Hospital of Shenzhen University, Shenzhen, Guangdong, People's Republic of China.

**Keywords:** scientometrics, knowledge mapping, scRNA-seq, musculoskeletal diseases, current frontiers, publication hotspots

## Abstract

**Background:** Worldwide, approximately 1.7 billion people are afflicted with musculoskeletal (MSK) diseases, posing significant health challenges. The introduction of single-cell RNA sequencing (scRNA-seq) technology provides novel insights and approaches to comprehend the onset, progression, and treatment of MSK diseases. Nevertheless, there is a remarkable lack of analytical and descriptive studies regarding the trajectory, essential research directions, current research situation, pivotal research focuses, and upcoming perspectives. Therefore, the aim of this research is to present a comprehensive overview of the advancements made in scRNA-seq for MSK disorders over the past 15 years.

**Methods:** It utilizes a robust dataset derived from the Web of Science Core Collection, encompassing January 1, 2009, through September 6, 2024. To achieve this, advanced analytical methodologies were applied to conduct thorough scientometric and visual analyses.

**Results:** The findings underscore the preeminent role of China, which contributes 63.49% of the total publications, thereby exerting a substantial impact within this research domain. Notable contributions came from institutions such as Shanghai Jiao Tong University, Sun Yat-sen University, and Harvard Medical School, with Liu Yun being the leading contributor. *Frontiers in Immunology* published the greatest number of research papers in this field. This study identified joint diseases, bone neoplasms, bone fractures, and intervertebral disc degeneration as the main research focuses.

**Conclusion:** This extensive scientometric analysis provides substantial benefits to both experienced and novice researchers by facilitating immediate access to critical data, thereby fostering innovation within this field.

## Introduction

Musculoskeletal (MSK) diseases significantly impact disability-adjusted life years, affecting an estimated 1.7 billion people worldwide with conditions such as degeneration, fractures, and other orthopaedic problems^1^-^6^. Resulting from processes like aging, injury, and immune system dysregulation, these disorders affect various MSK components, including bones (e.g., osteoporosis (OP), osteopenia), articular structures (e.g., osteoarthritis (OA), rheumatoid arthritis (RA)), the vertebral column (e.g., degenerative disc disease, ankylosing spondylitis), and muscular tissues (e.g., sarcopenia)^7^. These disorders significantly impair patients' work capacity and life quality, concurrently exerting considerable strain on the global healthcare infrastructure^8^, ^9^. Therefore, a comprehensive exploration of the etiology and treatment methods for these conditions is urgently needed. Advanced high-resolution research techniques are essential for the precise elucidation of the underlying mechanisms and the development of more efficacious treatments for MSK disorders^10^.

**Figure 1:**
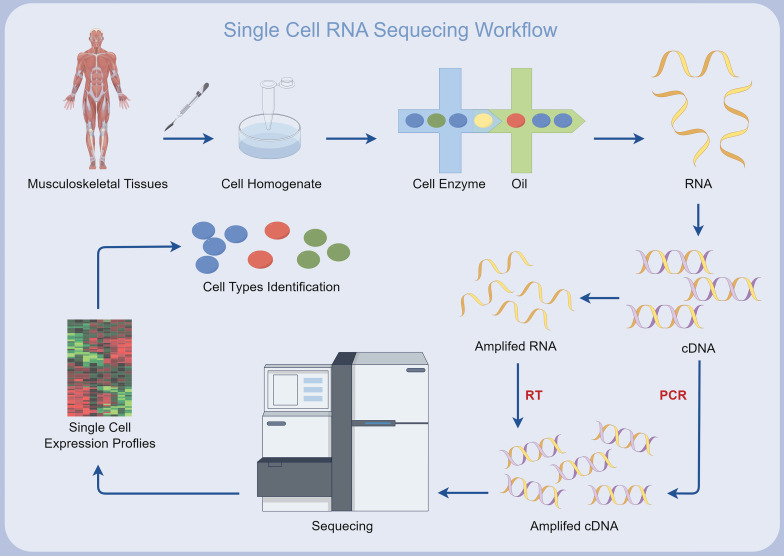
Simplified workflow of single-cell RNA sequencing for musculoskeletal diseases, created using Figdraw (https://www.figdraw.com/static/index.html#/).

In the last decade and a half, single-cell RNA sequencing (scRNA-seq) has become an indispensable technique for detailed transcriptome analysis at the cellular scale^10^-^15^. Contrasting with bulk RNA sequencing, which amalgamates gene expression data from multitudinous cells, scRNA-seq constructs sequencing libraries that correlate individual cell transcriptomes, thereby delineating cellular heterogeneity^16^, ^17^. Owing to its high-throughput nature, scRNA-seq facilitates comprehensive genetic profiling, capable of analyzing more than 10^6^ single cells within a singular experimental setup. This potentially reveals new cell types and defines the molecular roles within various cellular subpopulations^18^. Recently, scRNA-seq has been widely applied in the study of MSK disorders, uncovering previously unknown aspects of the MSK microcosm^10^, ^19^ (Figure 1). These approaches have provided an unprecedented level of examination of MSK systems, enhancing our understanding of cellular diversity and the key processes that regulate MSK equilibrium and pathogenesis^20^-^23^. Moreover, scRNA-seq has elucidated the intricate molecular networks governing intercellular communication, offering vital insights into the cellular microenvironments that frequently modulate disease states^24^-^28^.

Previous reviews have examined the application of scRNA-seq in MSK diseases from different viewpoints^10^, ^24^, ^29^-^36^, However, these analyses frequently suffer from a dearth of empirical backing via objective visual data, predominantly depending on subjective assessments constrained by their respective disciplinary frameworks. As a result, such reviews commonly exhibit inconsistencies and a pronounced subjective bias, which obstruct a thorough exploration and accurate delineation of the extant research landscape. Moreover, they make it difficult to identify research focus points and emerging trends. To surmount these limitations, this study uses scientometric analysis to graphically depict the broad landscape of "scRNA-seq in MSK diseases" over the last 15 years. In contrast to traditional reviews, our investigation provides empirical evidence derived from objective visual data, significantly diminishing subjective biases and variability. This approach facilitates an exhaustive analysis and affords a definitive depiction of the prevailing research landscape. Furthermore, our study augments the current body of literature by delivering an intricate data analysis and visualization, employing advanced tools like CiteSpace and VOSviewer.

This investigation seeks to elucidate the following research questions (RQs): RQ1: What are the prevalent trends in applying scRNA-seq to MSK disorders? RQ2: Which nations, academic entities, and researchers wield considerable influence in this domain? RQ3: What constitute the pivotal publications, critical references, and significant keywords? RQ4: Which diseases are primarily associated with this scholarly endeavor? This study employs a mixed-methods approach, integrating both quantitative and qualitative analyses. Quantitative data encompasses research themes, publication chronology, linguistic diversity, focus of journals, and comprehensive demographic details regarding authors, nations, institutions, journals, and citation indices. Qualitative evaluations are dedicated to the systematization of keywords. The development of an exhaustive and systematic repository of knowledge not only assists multidisciplinary researchers but also furnishes essential orientation for novices in pinpointing fertile areas of investigation. To the best of our knowledge, this represents the inaugural scientometric review specifically targeting the application of scRNA-seq in MSK disorders.

## Materials & Methods

### Data Source & Retrieval Strategy

The Web of Science Core Collection (WoSCC) (https://www.webofscience.com/wos/) was chosen as the principal data source for this scientometric analysis, given its exceptional comprehensiveness and accuracy, which are particularly advantageous for investigating the multidisciplinary domain of scRNA-seq in MSK diseases. The interdisciplinary nature of scRNA-seq research, spanning fields such as clinical medicine and molecular biology, necessitates the use of an integrated database capable of facilitating a broad yet in-depth analysis. Moreover, the WoSCC offers detailed citation records that are indispensable for constructing knowledge maps^37^, enabling a nuanced exploration of the interconnected research landscape. Its citation reporting functionality further serves as a validation mechanism, ensuring reliability and robustness in the scientometric evaluation. An additional benefit of the WoSCC lies in its advanced scientometric tools, which allow precise categorization of documents and reduce data inconsistencies, thus safeguarding analytical integrity^38^, ^39^. Furthermore, the inclusion of the Science Citation Index Expanded in the WoSCC enables the tracking of scientific advancements and the analysis of publication patterns^38^, ^40^-^42^, meeting stringent quality assurance criteria. Finally, the WoSCC's adherence to scientometric principles, such as Bradford's and Garfield's Laws^37^, ensures comprehensive coverage of core literature, thereby minimizing the likelihood of omissions and enhancing the dependability of the results. These attributes collectively establish the WoSCC as a leading choice for scientometric research.

In this study, we performed a comprehensive online search through the WoSCC to identify original research articles and review papers pertaining to scRNA-seq in MSK disorders. Starting from the work by Tang *et al.* in 2009^11^, who first used single-cell high-throughput sequencing for mRNA transcriptome profiling in mammalian cells, our search covered publications from January 1, 2009, to September 6, 2024. We used Medical Subject Headings (MeSH) and free words. The search protocol underwent iterative refinement under the guidance of three researchers to improve its sensitivity and specificity. A comprehensive description of the methodology is provided in the Supplementary Materials.

### Inclusion & Exclusion Criteria

The inclusion criteria for this investigation were confined to studies employing scRNA-seq to explore MSK disorders, specifically focusing on original research articles and review articles published in English. The exclusion criteria encompassed dissertations, personal correspondences, editorials, commentaries, conference abstracts, and studies featured in journals with irregular naming conventions. The research team engaged in thorough deliberations to establish the definitive inclusion and exclusion criteria.

### Scientometric Visualization & Data Analysis

Data were collected from the WoSCC database and imported into WPS Office 12.1.0 (Kingsoft Office, China). Subsequently, the analysis was conducted using the following tools: VOSviewer 1.6.18 (Leiden University, Netherlands), CiteSpace 6.3.R1 (Chaomei Chen, China), Pajek 5.16 (University of Ljubljana, Slovenia), Scimago Graphica 1.0.35 (https://www.graphica.app/, USA), and the chorddiag R package (R Studio, version 4.2.0).

Chord diagrams illustrating country/region cooperative relationships were created with VOSviewer and the chorddiag R package. The concurrence of nations/regions, academic institutions, contributors, periodicals, disciplines, keywords, and illnesses was analyzed using VOSviewer, Pajek, and Scimago Graphica software. A visual inspection of these entities and co-citation networks in the literature was carried out using Citespace, resulting in the creation of relevant graphical representations. To clarify the most influential citation trends, burst detection graphs were made, highlighting the top 10 citation surges for nations/territories, academic institutions, contributors, and key terms, as well as the top 20 for co-cited literature.

Information pertaining to diseases was sourced from the Citexs Data Analysis Platform, accessible at https://www.citexs.com. This platform provides a powerful set of tools for creating visual representations, which is crucial for conducting an in-depth analysis of the current conditions, key areas of interest, and potential developments in the field.

## Results & Discussion

### Scientific Output

**Figure 2:**
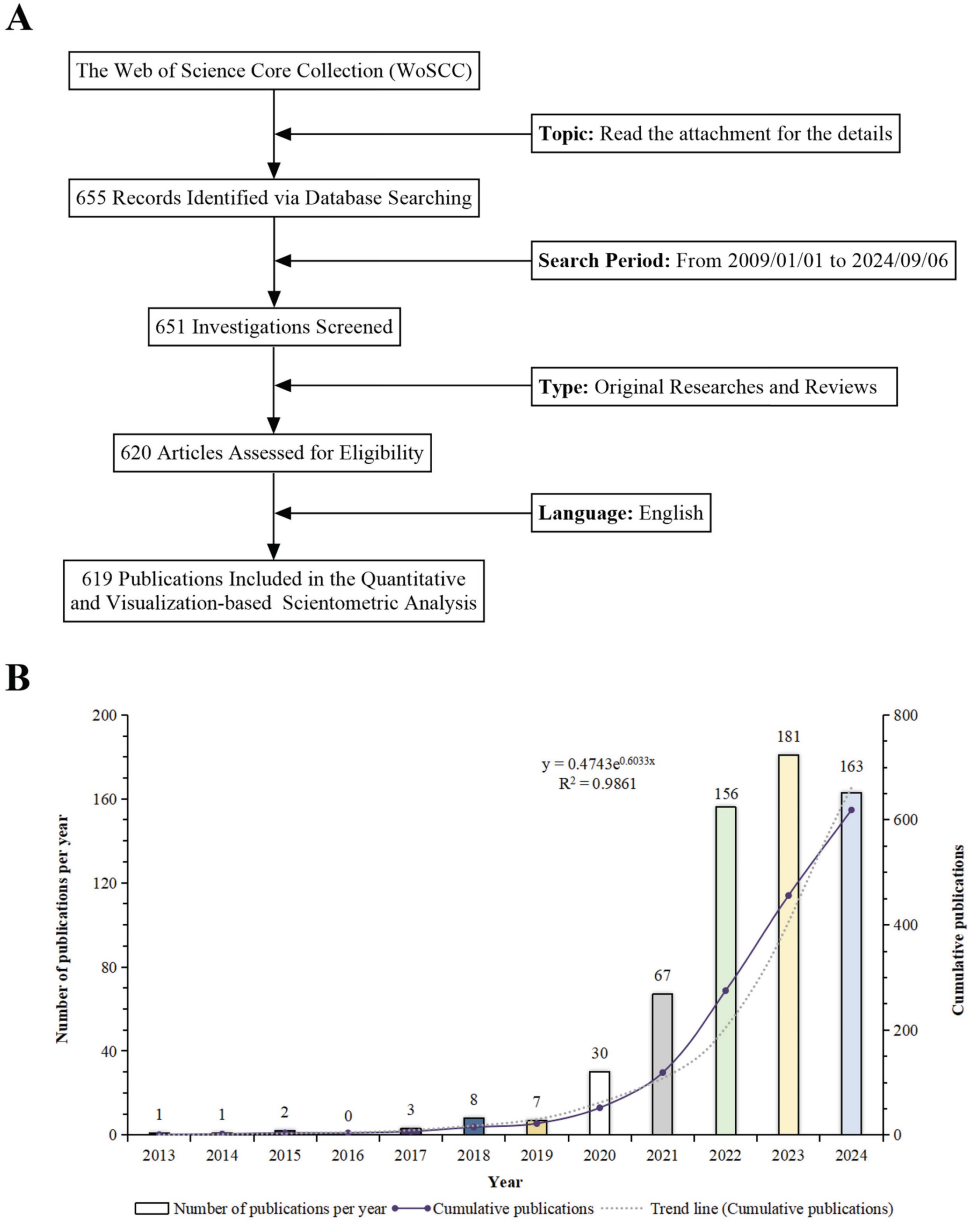
(A) Schematic diagram of the literature search and selection methodology. (B) Temporal trend analysis of research on "single-cell RNA sequencing in musculoskeletal diseases" from 2009 to 2024.

Although our search covered the period from January 1, 2009, to September 6, 2024, the earliest retrieved paper dates back to 2013. Thus, all subsequent data analysis pertains to the period from 2013 to September 6, 2024. The process of data acquisition and compilation is depicted in Figure 2A. The progression of the study is illustrated through the systematic aggregation of relevant scientific literature. There was a collection of 619 relevant publications on "scRNA-seq in MSK diseases" from 2013 to 2024. The dataset comprises 534 original articles and 85 review papers, yielding an average of 56.27 publications per year. This trend underscores the substantial attention and interest garnered by the field. Beginning in 2022, annual publications surpassed 150, culminating in a peak of 183 publications in 2023. The graphical representation indicates an impressive 181-fold increase in the number of publications since 2013, highlighting both the marked escalation in research activity and the increasing prominence of this research domain. An exponential growth model, defined as y = 0.4743e^0.6033x^, where the year is x and y represents the publication count, closely matches the empirical data. It has an outstanding coefficient of determination (R²) of 0.9861. This model showcases the accuracy of the data analysis presented in Figure 2B. The trend line predicts an upward trend in annual research, highlighting the growing interest in scRNA-seq for MSK disorders and suggesting substantial future progress in the field.

The exponential growth in scRNA-seq research for MSK diseases underscores the need for robust funding policies and enhanced infrastructure to accelerate progress. Policymakers can leverage this data to expand resource allocation, foster regulations that enhance collaborative research, and promote the development of targeted therapies to improve public health. This trend marks a dynamic field of study, presenting extensive opportunities for new discoveries and interdisciplinary collaborations. Researchers can utilize the expanding knowledge base to investigate less-studied aspects of MSK diseases, potentially yielding groundbreaking treatments and diagnostics. The high R^2^ value of the predictive model shows the robustness of the research data, providing a reliable basis for future studies and hypotheses. The surge in publications and keen interest suggests a mature market for developing and implementing advanced diagnostic technologies. Specialized biotech and pharmaceutical companies can capitalize on these findings by investing in targeted research and development to commercialize scRNA-seq applications. The predictive model indicates continued interest, highlighting its potential as a promising area for long-term investment.

### Performance Analysis

#### Countries/Regions

**Figure 3:**
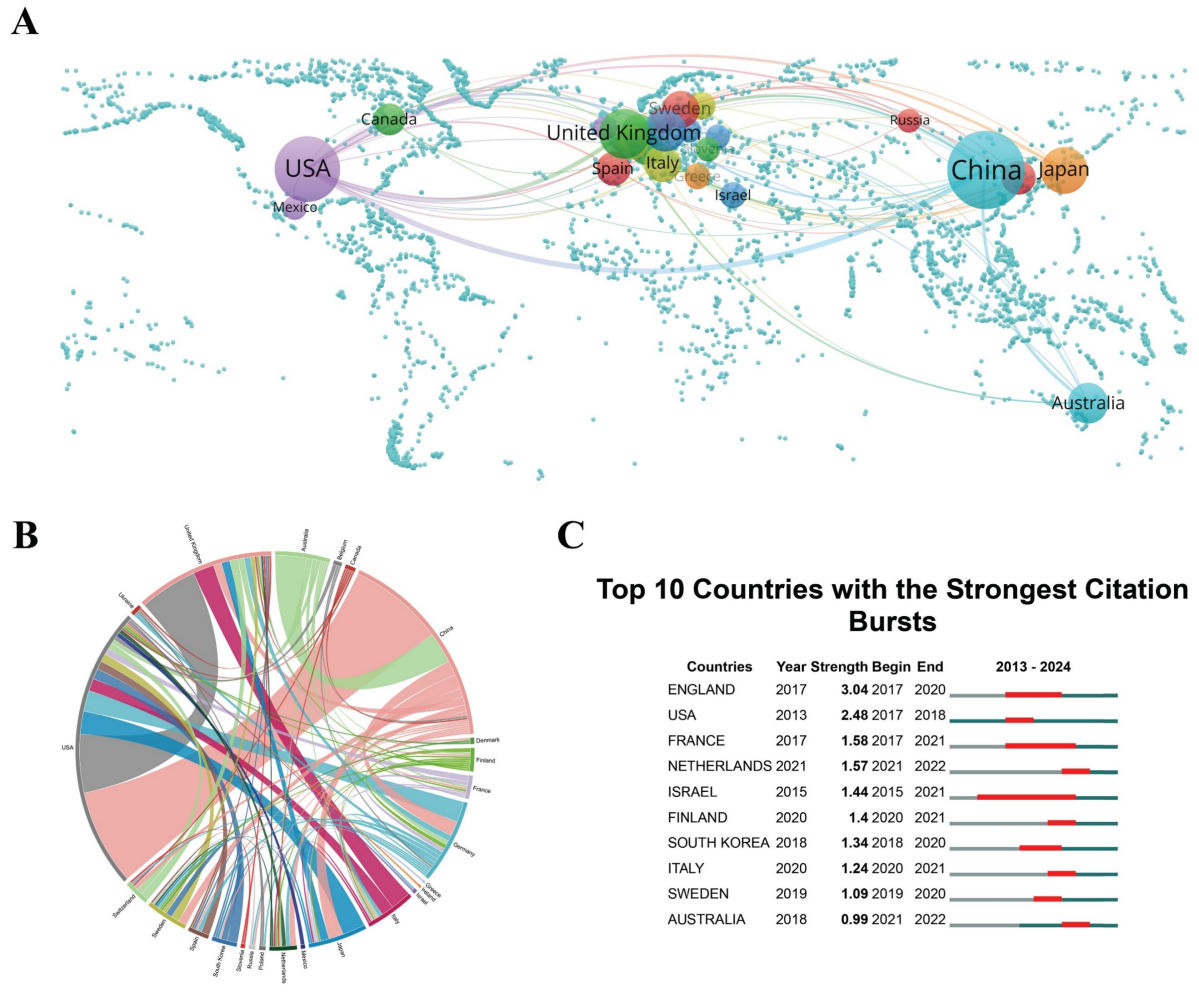
(A) Global map of single-cell RNA sequencing (scRNA-seq) research in musculoskeletal (MSK) diseases, where spheres represent countries. The size of each sphere indicates the number of publications, while the line thickness reflects the collaboration intensity between countries. (B) Chord diagrams depicting international collaborations in scRNA-seq research on MSK diseases. Line thickness represents collaboration strength between countries. (C) Research output from the top 10 countries on scRNA-seq in MSK diseases, highlighted in red to denote increased publication volume. "Burst" describes a rapid rise in research activity, with "BurstBegin" and "BurstEnd" marking its start and decline, respectively.

Research on "scRNA-seq in MSK diseases" encompasses contributions from 43 countries/regions. Figure 3A and 3B visualize the international collaboration networks, highlighting the contributions of countries with a minimum of six publications. These findings provide valuable insights for fostering strategic partnerships and promoting the effective dissemination of knowledge^43^.

China, with a contribution of 393 publications, represents 63.49% of the overall research output, which is more than 15 times the volume of Germany's contributions, underscoring its central role in propelling the research on scRNA-seq in MSK disorders. China's leadership in scRNA-seq MSK research is partly due to significant investments in research infrastructure and technology. Meanwhile, China, along with the United States and the United Kingdom, has numerous academic institutions that prioritize cutting-edge research and development in life sciences, which sustains a steady output of high-quality research. These advancements facilitate large-scale studies that contribute substantially to the field's body of knowledge. The United States and the United Kingdom contribute significantly, with 175 and 52 publications respectively, representing 28.27% and 8.40% of global research in this field. High publication and collaboration rates in key regions suggest potential markets for the clinical applications of scRNA-seq technologies, particularly relevant for biotech and pharmaceutical sectors. This information may guide decisions on focusing product development and marketing strategies. The concentration of research in China, the United States, and the United Kingdom suggests that policymakers should consider strategic investments in scRNA-seq research to sustain or improve their competitive advantage. The extensive collaboration among these nations indicates the benefits of fostering international partnerships, which could lead to global guidelines and funding initiatives to support ongoing research and collaboration.

In the chord diagram, the peripheral curve segments correspond to different countries or regions, with the length of each segment proportional to the respective number of publications originating from that area^44^. National collaboration levels are shown through the connectivity in the diagram. The cooperation intensity between China and the United States is the highest among country pairs (link strength = 46), indicating frequent and close collaboration. Next, the link strength between the United States and the United Kingdom is 28, and that between Australia and China is 15 (Figure 3B). The high connectivity and collaboration rates between China and countries like the United States and the United Kingdom reflect strong international agreements and partnerships, possibly encouraged by governmental and institutional policies that support collaborative international research. For researchers, these networks provide critical insights that facilitate the formation of strategic alliances^45^. The dense collaboration networks highlight strategic alliances that likely speed up knowledge exchange and innovation, contributing to the rapid progress in the field. Understanding key players and their relationships helps establish productive partnerships, select impactful conferences, and identify potential mentors and collaborators.

Citation bursts help identify which research has had the most significant impact, guiding companies and research institutions in targeting areas of major breakthroughs. This information is vital for recruiting talent and making informed investments in emerging fields. Figure 3C presents the citation bursts for the top 10 countries/regions, with the intensity of each burst denoted by red lines. Notably, England experienced a marked surge in citations (burst strength = 3.04) from 2017 to 2020, followed by the United States (burst strength = 2.48) and France (burst strength = 1.58), reflecting their increasing prominence within this research domain. The citation bursts observed in England, the United States, and France indicate that these regions are producing highly influential research contributions. These citation bursts indicate where influential research is emerging, advising companies on where to focus for major advancements or recruitment.

The predominance of certain countries in scRNA-seq research for MSK diseases underscores their capabilities and strategic orientations, while also raising questions about the global scalability and applicability of the research outcomes. Policymakers in less represented countries may struggle to align their healthcare policies with findings from dissimilar health ecosystems. Addressing these issues necessitates efforts to enhance the geographical diversity of research, ensuring the global applicability and inclusiveness of health advancements. For the biotech and pharmaceutical industries, concentrated research output and effective collaboration highlight strong regions for market entry and product development. However, this concentration may create competitive market conditions, potentially challenging for new entrants.

#### Institutions

Researchers can gain significant insights into potential collaborators and prominent institutions in the "scRNA-seq in MSK disorders" field. By visualizing the density of collaborations and cluster formation, areas of synergy and expertise can be identified, which is helpful for making informed decisions about research partnerships and projects. Policymakers can use the findings from the analysis of collaborative networks to identify leading institutions and the geographical distribution of research efforts. Such insights can guide strategic investments and policy formulation to support impactful research and foster global collaborations in the "scRNA-seq in MSK disorders" field. Industry professionals can strategically align with these leading institutions by understanding the hotspots of significant research and innovation. This alignment can facilitate product development, technology licensing, and co-funding of research, thereby turning scientific breakthroughs into market-ready solutions.

**Figure 4:**
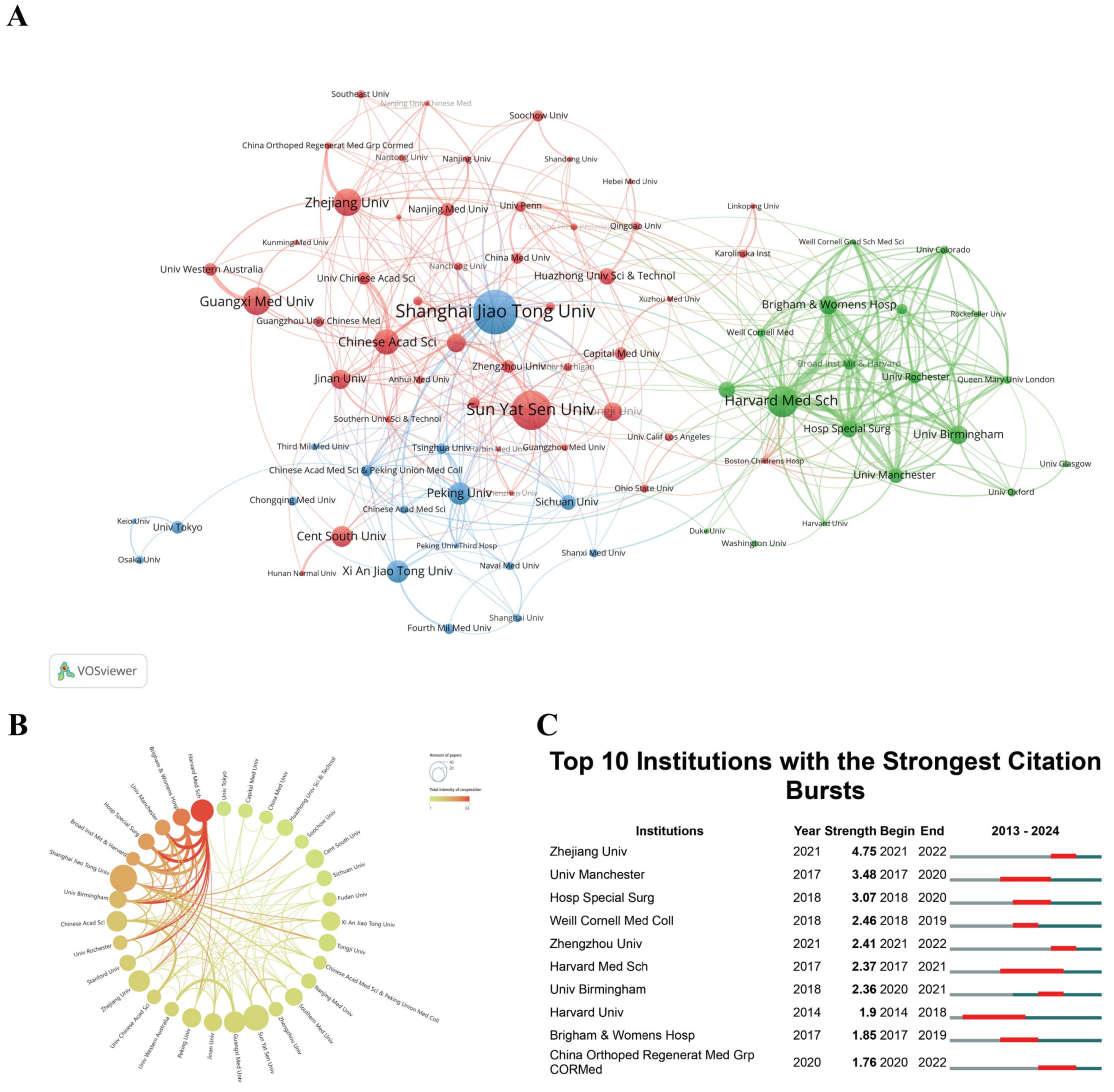
(A) Hierarchical clustering diagram of research institutions based on co-citation networks. Distinct colors identify different clusters, with line thickness representing cooperation strength and circle size indicating the number of publications per institution. (B) Diagram showing the intensity of institutional cooperation. Line thickness reflects the strength of collaboration, while circle size represents the number of publications from each institution. (C) Citation bursts in the top 10 institutions, marked by red bars for periods of heightened citation activity. "Burst" indicates a rapid rise in a topic's prominence, with "BurstBegin" and "BurstEnd" denoting the start and decline of this growth, respectively.

The research institution cooperation and clustering maps, shown in Figure 4A and 4B, were generated by using a minimum publication threshold of 10 and 5 documents per institution respectively. The distinct colors of the regions represent different clustering patterns. The thickness of the lines connecting the circles reflects the intensity of collaboration between institutions, while the size of each circle is proportional to the number of documents published by the respective organization. In the past 11 years, the global research landscape for "scRNA-seq in MSK diseases" has grown significantly, involving 981 entities. The leading institutions were Shanghai Jiao Tong University (n = 38, 6.14%), followed by Sun Yat-sen University (n = 34, 5.49%), and Harvard Medical School (n = 27, 4.36%). These institutions represent potential partners for future research, offering opportunities for collaborative initiatives and knowledge exchange. In the realm of institutional partnerships, Harvard Medical School has emerged as a prominent collaborator, underscoring a strong commitment to fostering alliances with other institutions. This commitment is exemplified by the substantial affiliations between Harvard Medical School and other leading academic organizations.

Understanding institutions experiencing citation bursts is essential for both researchers and industry professionals. Identifying the sustained impact and adaptability of research initiatives provides a comprehensive evaluation of an institution's scholarly contributions^46^. Through CiteSpace analysis (Figure 4C), this study has identified institutions that have exhibited significant citation surges. Zhejiang University took the lead, with a citation burst from 2021 to 2022 (strength = 4.75). Harvard University had the longest citation burst from 2014 to 2018. Unfortunately, this trend has not persisted in the past six years. On the contrary, Zhejiang University, Zhengzhou University, and China Orthopedic Regenerative Medicine Group had a citation surge from 2020 to 2022. This increase can be partly attributed to substantial investments in biomedical research by the Chinese government, aiming to make China a leader in global scientific research. The establishment of high-throughput genomic centers in these universities has created a strong research environment conducive to large-scale studies and innovations.

#### Authors

**Figure 5:**
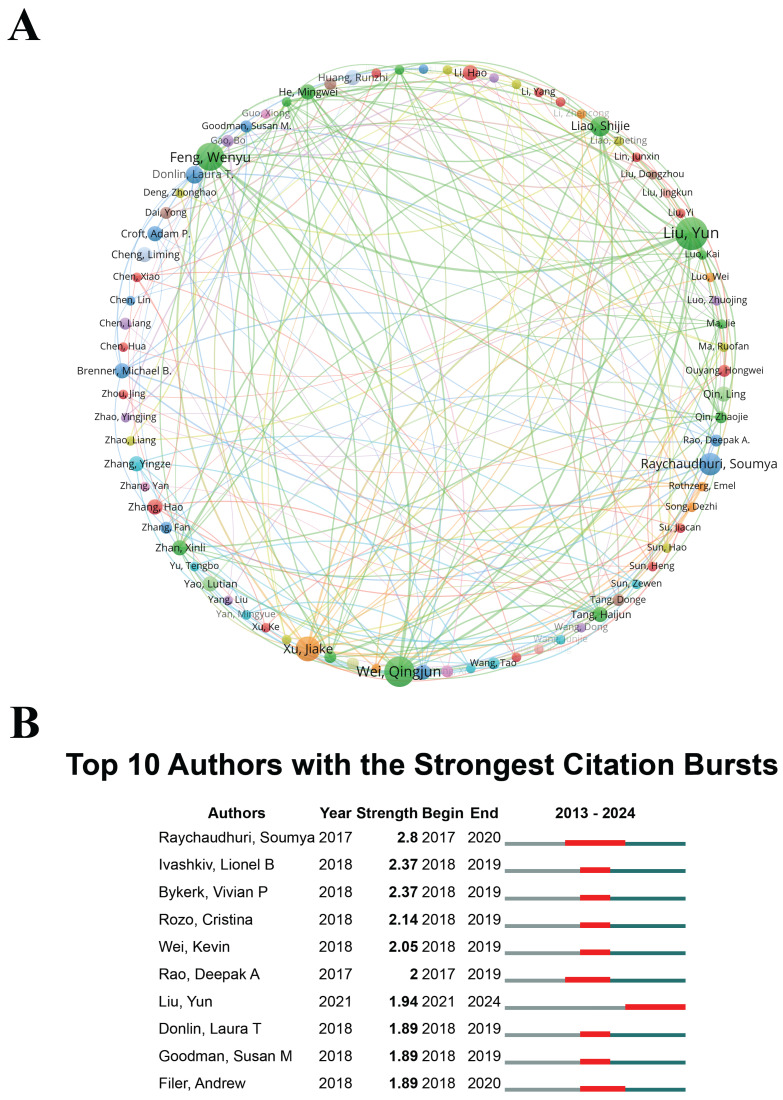
(A) Author co-occurrence map, where each node consists of a circle and a text label, and different colors indicate distinct clusters. (B) Top 10 authors exhibiting the most significant citation bursts in studies on "single-cell RNA sequencing in musculoskeletal diseases". "Burst" indicates a rapid escalation in research interest within a particular period, with "BurstBegin" denoting its initiation and "BurstEnd" its decline.

Identifying leading experts and analyzing their collaborative networks is instrumental in recognizing key contributors within the field. Experts distinguished by high citation rates and a consistent publication record offer critical insights that can influence the trajectory of future research. Through a comprehensive analysis of authorship within the domain of "scRNA-seq in MSK disorders", this study identified 4,654 scholars as significant contributors. Among them, 80 researchers were distinguished by their prolific output, each authoring at least four publications. These scholars are likely recognized experts with substantial expertise and influence, making their insights invaluable to researchers in the "scRNA-seq in MSK disorders" field. To further examine collaborative patterns, VOSviewer software was employed to generate visual diagrams, using a minimum publication threshold of four per author. The visualizations depict node sizes proportional to each author's publication count, with distinct colors representing different author clusters. The thickness of the links between nodes reflects the strength of collaborative interactions. Liu Yun and Wei Qingjun were particularly prominent for their collaborative ties, as shown in Figure 5A. Additionally, Liu Yun (2.10%, 13 publications), Wei Qingjun (1.94%, 12 publications), and Feng Wenyu (1.78%, 11 publications) have been recognized as prominent scholars in the field of "scRNA-seq in MSK disorders", underscoring their significant contributions to advancing this area of research.

The analysis of citation bursts serves as an essential indicator, reflecting the frequency with which an author's work is referenced within a particular academic discipline over a specified timeframe. Figure 5B enumerates the top ten authors who have accrued the highest citation counts within the domain of "scRNA-seq in MSK disorders". Notably, Soumya Raychaudhuri experienced a pronounced citation burst between 2017 and 2020, achieving the highest burst strength value of 2.8. The initiation of citation bursts for these top ten authors post-2017 underscores an escalating scholarly interest in "scRNA-seq in MSK disorders" research, spanning from 2017 through 2024. Notably, Soumya Raychaudhuri and Liu Yun had the longest citation burst periods, lasting 3 years, signifying that their research remains highly valued and has a lasting academic impact in the field. The groundwork established by these distinguished scholars creates a robust framework for future investigations in this field, guaranteeing that forthcoming studies leverage the influential and extensively cited body of existing research. Furthermore, these eminent scholars offer crucial mentorship and direction to new researchers delving into this specialized domain. Moreover, their significant contributions can offer valuable insights for professionals, highlighting key figures and emerging trends that may influence their collaborative efforts and strategic planning.

Policymakers can use the insights from leading experts' citation rates and publication records to identify research areas requiring further support and development. Understanding who the key contributors are and their collaboration networks can inform funding allocation, policy formulation, and the creation of initiatives aimed at enhancing research capabilities in this field. For researchers, knowing the leading figures in the field and their collaboration patterns helps identify potential mentors, collaborators, and sources of inspiration. The demonstrated citation bursts indicate enduring influences, guiding researchers towards impactful and relevant topics and methodologies that have shaped the current landscape. Industry practitioners can leverage the expertise of these top scholars to guide product development and innovation strategies. Understanding the research trends and focal areas of these leading experts allows industry stakeholders to align their research and development efforts with cutting-edge discoveries, potentially leading to more effective and targeted therapies in MSK treatments.

### Journals, Related Fields, and Co-cited References

#### Journals & Related Fields

**Figure 6:**
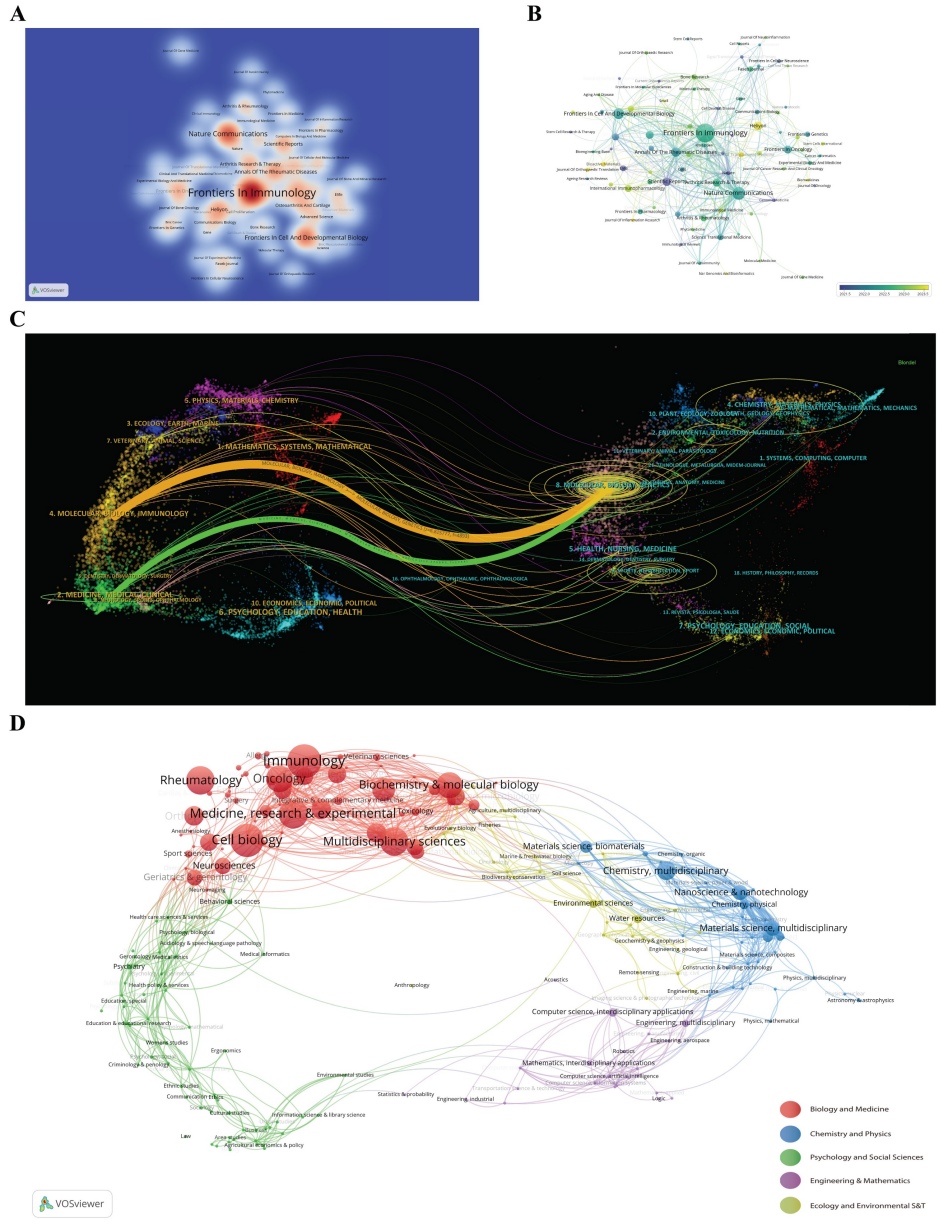
(A) Density visualization map showing journal citations, with color intensity indicating publication volume. (B) Journal distribution map categorized by average publication year, with blue indicating earlier and yellow later years. Node size represents keyword frequency, and circle color gradients indicate the publication year. (C) Dual-map overlay depicting citation networks among journals on single-cell RNA sequencing in musculoskeletal diseases. Points represent journals, and connecting curves illustrate citation links, revealing interdisciplinary trends and citation evolution. (D) Analysis of research subject areas, with different colored spheres representing various disciplines.

The visualization of journal publication data offers a comprehensive overview of the scholarly communication landscape across 256 journals that have published articles on scRNA-seq in MSK disorders. A heat-based graph, based on thermodynamic principles, is used to show document distribution, with a minimum requirement of four papers per journal for inclusion. The color intensity on the graph indicates the number of papers published by each journal (Figure 6A), helping researchers identify suitable journals for submission. *Frontiers in Immunology* leads with 46 publications, followed by *Nature Communications* and *Frontiers in Cell and Developmental Biology*, each with 19 publications. Identifying leading journals and understanding their citation metrics are essential for researchers aiming to evaluate the potential impact of their research and to choose suitable publication venues. Figure 6B uses a color-coding system to represent the founding years of journals, where each circle and its label forms a node. The size of each circle is proportional to the number of papers published by the journal. The color of each sphere corresponds to the average publication year, with blue representing earlier years and yellow signifying more recent years, as indicated by the color gradient in the lower right corner of the figure. The figure shows that among the top 20 journals, *Scientific Reports*, *eLife*, and *Osteoarthritis and Cartilage* published earlier, between 2021 and 2022, while *Heliyon*, *Advanced Science*, *International Immunopharmacology*, and *Bone Research* published more recently, indicating the recent focus and direction of these journals in this field.

The dual-map overlay of journals serves as an effective tool for illustrating the dynamic shifts in scientific research centers and the dispersal of journals across various academic disciplines^47^. The labels on the map delineate the research domains addressed by the articles within these journals. Journals that cite other scholarly works are positioned on the right, whereas those referencing additional sources appear on the left. Citation pathways are depicted through lines of varying colors, where the line thickness corresponds to citation frequency, measured on a *z*-score scale^38^. Such visualizations are instrumental in detecting emerging trends and directional shifts in scientific research, thereby aiding researchers in aligning their investigative pursuits more strategically. Figure 6C reveals that research on "scRNA-seq in MSK diseases" predominantly occurs within the domains of molecular biology, immunology, genetics, and clinical medicine. This highlights the interdisciplinary approach of the research and pinpoints the journals that are most pertinent to these areas. The interdisciplinary focus highlights the broad scientific impact of "scRNA-seq in MSK diseases" research, which integrates various knowledge domains to address complex health challenges. The frequency and intensity of citations reveal notable shifts in scientific emphasis, suggesting that research in "scRNA-seq in MSK diseases" is increasingly recognized across diverse disciplines. This trend may well influence future research trajectories and drive innovation within these fields.

The VOSviewer software was utilized to carry out a visual analysis of 619 scholarly articles, which were systematically classified into five main research areas. This systematic classification enhances our comprehension of the various research domains and their intricate interconnections, providing an essential resource for researchers and industry professionals engaged in the evolving field of "scRNA-seq in MSK diseases". The clustering analysis is depicted in Figure 6D, where spheres of varying colors denote distinct research areas. The findings disclose a notable emphasis on "Biology and Medicine" within the studies, with a considerable number of publications assigned to "Immunology", "Cell Biology", "Rheumatology", and "Multidisciplinary Sciences".

These results are crucial for directing stakeholders in strategically navigating the dynamic landscape of scRNA-seq research within the field of MSK diseases. This alignment supports the broader goal of maximizing research impact and applications to enhance health outcomes and advance scientific knowledge. Policymakers can utilize this data to comprehend research output distribution and identify leading journals, guiding funding decisions and policy development. Insights into emerging trends and journal focuses enable strategic resource allocation to support promising research areas. Researchers benefit from these visualizations by identifying leading journals in their field, which aids in strategically submitting their work. Understanding citation pathways and emerging trends helps researchers align their work with the most impactful themes, optimizing their field contributions. Industry professionals can deduce which areas of "scRNA-seq in MSK diseases" are gaining traction, indicating potential market and innovation directions. Insights into predominant research themes and journals inform collaborative efforts, patent strategies, and product development, aligning with recent scientific advancements.

#### Co-cited References

**Figure 7:**
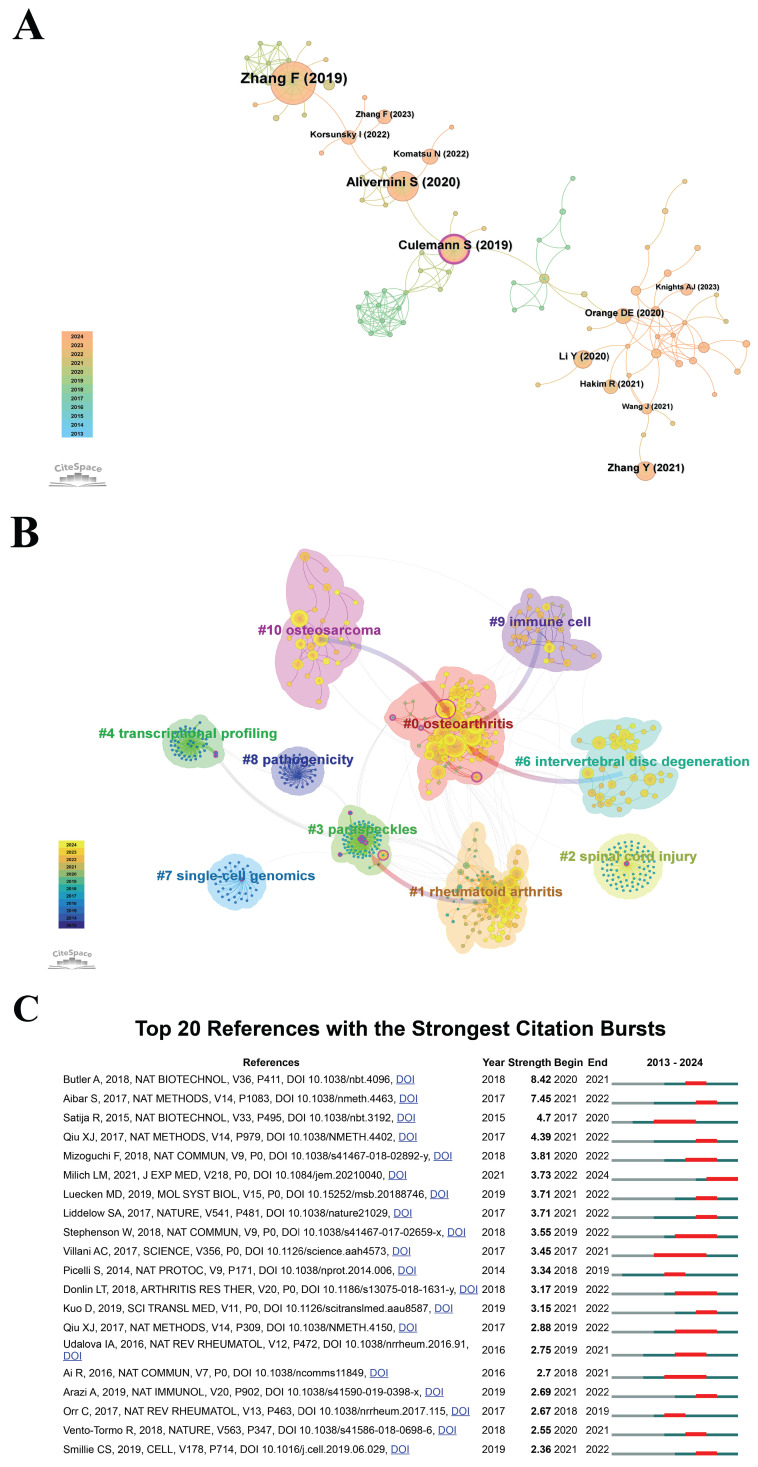
(A) Literature co-citation analysis diagram. The aggregate size of overlaid spheres, each representing an annual citation ring, correlates with the number of co-citations. Purple indicates earlier citations, yellow denotes later citations, and blended colors indicate citations across these years. Lines connecting the spheres depict co-citation links, with key nodes highlighted in rose red. (B) Cluster analysis diagram of literature co-citation. The total size of the spheres, representing annual citation data, is proportional to the volume of co-citations. Purple indicates earlier citation periods, yellow shows later periods, and mixed colors mark citations throughout these times. Lines between spheres illustrate the co-citation network, with central nodes in rose red indicating a centrality score above 0.1. (C) Top 20 references identified by their significant citation bursts. A "burst" denotes a sharp increase in prominence of a research topic within a defined period. "BurstBegin" signifies the start of this intense growth, and "BurstEnd" indicates the end of this growth phase.

The recognition of highly cited and pivotal publications is fundamental for researchers to comprehend the core aspects of their discipline and guide their investigative pursuits effectively. Figure 7A presents a co-citation network analysis of scholarly articles concerning "scRNA-seq in MSK diseases", spanning from January 1, 2013, to September 6, 2024, utilizing the CiteSpace tool. In this visualization, the diameter of each circle correlates with the volume of co-citations, transitioning in color from purple, indicating older articles, to yellow, denoting more recent works. The blending of these colors reflects a continuous citation trend over time. The lines interlinking the circles depict the co-citation linkages among the publications. Nodes accentuated in pink signify central nodes within the network, marked by a centrality score above 0.1, underscoring their pivotal role in academic discussions. The most extensively cited article, "Defining inflammatory cell states in rheumatoid arthritis joint synovial tissues" by Zhang Fan *et al.*, appeared in *Nature Immunology* in 2019 and has accumulated the highest co-citation count of 87^48^. This pioneering study in scRNA-seq research laid a foundation for subsequent investigations. Identifying influential publications is crucial for researchers aiming to deepen their grasp of the core concepts and methodologies propelling the field. This enhanced understanding allows researchers to expand upon established knowledge, investigate emerging trends, and focus on critical areas of inquiry. The consistent citation of these works, as denoted by the blended colors on the visualization circles, underscores their persistent relevance and impact in guiding contemporary research endeavors. This ongoing relevance is a testament to their seminal contributions to the field, providing a solid foundation for future scholarly work.

CiteSpace employs Modularity (*Q* value) and Mean Silhouette (*S* value) metrics to assess the integrity of network structures and the distinctiveness of cluster formations. A *Q* value exceeding 0.3 signifies substantial clustering, while an *S* value greater than 0.5 indicates clearly delineated clusters. The analysis executed returned a *Q* value of 0.8205 and an *S* value of 0.9557, both considerably surpassing these benchmarks, thereby verifying the robustness of the clustering structures identified. These findings substantiate the dependability of the clustering methodology implemented in this study. The analysis revealed 10 distinct clusters, as shown in Figure 7B: #0 osteoarthritis, #1 rheumatoid arthritis, #2 spinal cord injury, #3 paraspeckles, #4 transcriptional profiling, #6 intervertebral disc degeneration, #7 single cell genomics, #8 pathogenicity, #9 immune cell, and #10 osteosarcoma.

CiteSpace was employed to examine citation bursts within the research domain of "scRNA-seq in MSK diseases". Figure 7C illustrates the influence of the top 20 references, underscoring the substantial academic attention garnered by these publications. Butler Andrew *et al.*'s research in *Nature Biotechnology* has the highest citation burst intensity of 8.42, from 2020 to 2021^49^. Villani Alexandra-Chloé *et al.*'s research in* Science* had the longest citation burst, from 2017 to 2021, lasting four years^50^. The majority of the top 20 cited references have experienced significant increases in their citation counts from 2018 to 2024, reflecting a burgeoning interest in this research area since 2018. For scholars, the identification of such citation bursts serves as a crucial indicator for synchronizing their investigative pursuits with evolving scientific currents. Concentration on nascent themes enables researchers to maintain the relevance of their contributions to the scholarly dialogue within the domain of "scRNA-seq in MSK diseases". This strategic orientation not only propels advancements in the field but also enhances the application of research outcomes in clinical settings and policy formulation. Additionally, industry professionals can leverage insights gleaned from citation trends and co-citation networks to shape their investment decisions and product innovation strategies. By aligning with these emergent research currents, practitioners can capitalize on new opportunities and sustain a competitive stance in the rapidly evolving sector of "scRNA-seq in MSK diseases".

These findings serve as an essential navigational tool for stakeholders, enhancing strategic planning and execution in research, policymaking, and industrial innovation within the "scRNA-seq in MSK diseases" domain. Policymakers can use insights from co-citation networks and citation bursts to pinpoint research areas gaining momentum that require support or regulation. Understanding central topics in the field shapes health policies, funding priorities, and program development to address pressing MSK health challenges. Researchers significantly benefit from recognizing influential works and current citation trends. This knowledge directs them to foundational studies and emerging areas, aligning their efforts with the field's most impactful and innovative aspects. This alignment enhances the relevance of their work and boosts their contributions to the field's advancement. Industry professionals can leverage data from citation trends and key publications to inform their research and development strategies. Insights into areas receiving increased attention and innovation enable targeting development efforts towards the most promising and market-relevant technologies, aligning better with clinical needs and commercial potential.

### Keywords

**Figure 8:**
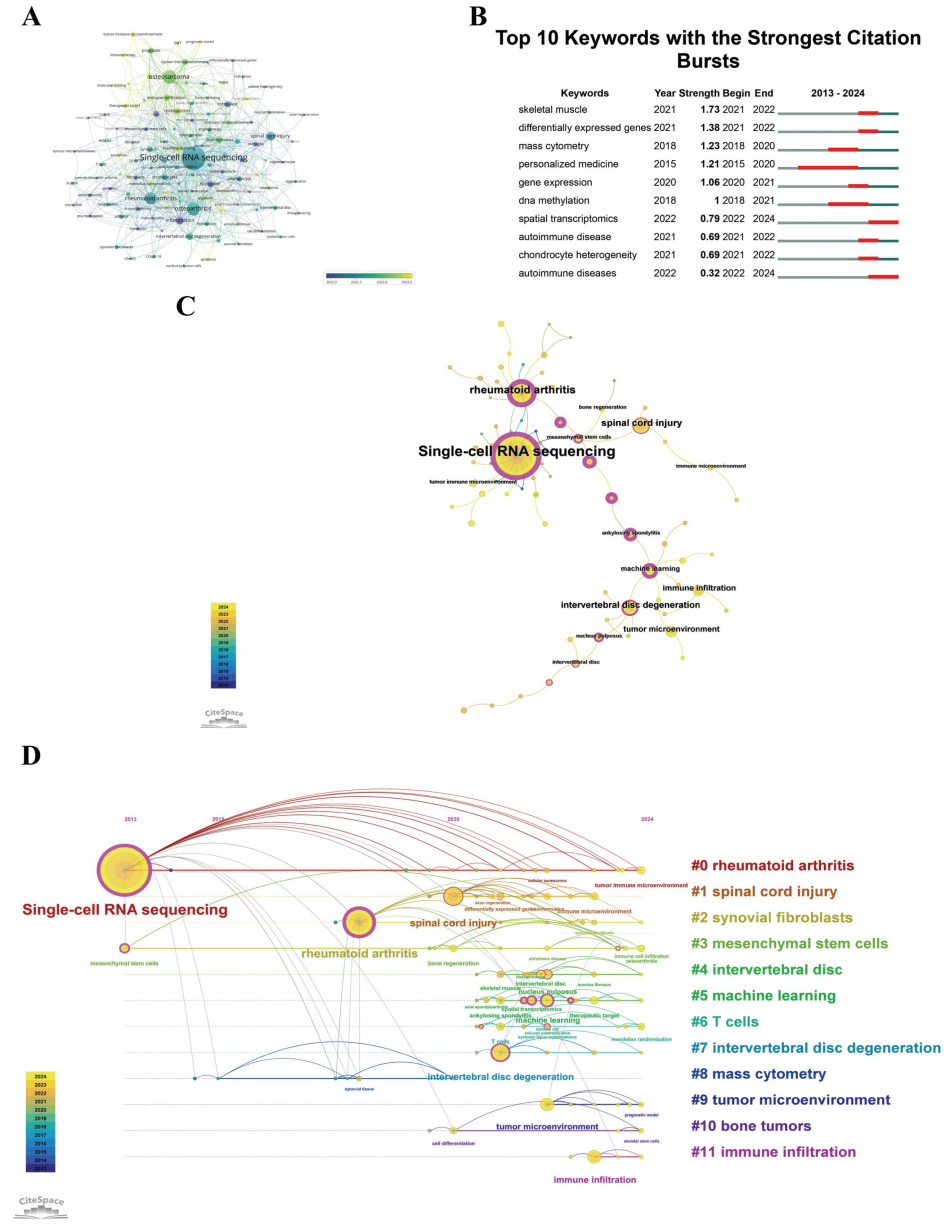
(A) Time-based visualization of keyword intensity, with nodes represented by circles whose size reflects keyword frequency. The color gradient from blue to yellow indicates the transition from early to recently emerged keywords, suggesting new research trends. (B) CiteSpace identified top 10 keywords exhibiting the strongest citation bursts. A "burst" signals a sharp rise in topic prominence, with "BurstBegin" and "BurstEnd" indicating its start and eventual tapering off, respectively. (C) Keyword co-occurrence analysis graph. The total size of overlaid spheres, representing annual citation data, correlates directly with keyword frequency. Purple spheres denote earlier emergence of keywords, yellow for later emergence, and blended colors reflect occurrence throughout the years. Lines between spheres show keyword co-occurrence, with key nodes highlighted in rose red. (D) Keyword clustering timeline analysis graph. Each sphere's size, corresponding to annual data, matches keyword frequency; connections between spheres denote keyword co-occurrence. Purple suggests earlier keyword emergence, yellow for later, and mixed colors denote continuous relevance. Rose red nodes indicate key central nodes. Keywords within the same cluster align horizontally, with initial emergence times at the view's top, moving rightward. This layout allows visualization of keyword volume and temporal distribution per cluster, indicating cluster significance and duration.

VOSviewer was utilized to conduct a clustering analysis, predicated on keyword co-occurrence, with inclusion criteria set at a minimum of three occurrences. The visualization exclusively depicted keywords satisfying this criterion, selected from an aggregate of 1,191 distinct keywords. Figure 8A displays a network encompassing 115 such keywords. This figure also illustrates the temporal dynamics in keyword usage frequency. Each node in the network is denoted by a circle, which is labeled with the respective keyword. The diameter of each circle reflects the frequency of the keyword, with larger circles denoting higher frequencies. The thickness of the lines interconnecting the circles indicates the robustness of the relationships between the keywords. The coloration of the circles signifies the average year of keyword emergence within the scholarly literature; blue signifies earlier emergence, whereas yellow denotes more recent appearances. The graph reveals that keywords such as "heterogeneity" and "inflammation" emerged early, around 2021~2022, while terms like "intervertebral disc degeneration", "macrophage", and "single-cell RNA sequencing" appeared in 2022. Keywords like "osteosarcoma", "immune infiltration", and "tumor microenvironment" emerged between 2023 and 2024, suggesting recent research focus areas. For researchers, keyword co-occurrence and burst analysis act as a roadmap, highlighting evolving areas of interest. This guidance informs their study designs and directs their focus towards topics with the potential for significant impact and collaborations.

Figure 8B prominently shows instances of keyword bursts that are associated with a significant increase in citations. Such an examination highlights the fields of study that are currently attracting considerable scholarly attention. The temporal analysis of keyword bursts is helpful in guiding strategic decisions about research investments and collaborative efforts. It ensures that these initiatives are in line with the latest research directions and promotes the establishment of strategic partnerships that are well-matched with the evolving landscape of scientific inquiry. As illustrated in Figure 8B, the term "skeletal muscle" exhibited the highest citation burst intensity, achieving a burst strength of 1.73 during the period from 2021 to 2022. "Personalized Medicine" had the longest citation burst duration, from 2015 to 2020, with citations increasing rapidly over these six years. Among the top 10 keywords, "spatial transcriptomics" and "autoimmune diseases" had citation bursts after 2023, indicating these as recent research hotspots and directions in this field. Policymakers can use keyword analysis to identify emerging trends and prioritize funding for rapidly growing research areas. This strategic focus helps the development of innovative treatments and technologies that are crucial for public health. Industry professionals can align their product development and innovation strategies with the identified key research areas. By understanding these trends, industry efforts stay market-relevant and in line with the latest scientific discoveries, enhancing both commercial and clinical value.

Co-occurrence analysis elucidates pivotal relationships among keywords, thus delineating the principal themes within the field. The software CiteSpace was employed to analyze the co-occurrence patterns of the keywords "scRNA-seq in MSK diseases" covering the period from January 1, 2013, to September 6, 2024, as depicted in Figure 8C. This visualization utilizes circles to denote the cumulative annual frequency of each keyword's utilization. Purple circles depict earlier occurrences, yellow circles signify more recent usage, and circles with blended colors indicate keywords that have been consistently referenced over multiple years. The connecting lines illustrate the co-citation relationships among the references. Magenta nodes highlight keywords that possess centrality scores exceeding 0.1, underscoring their significant role within the network. Figure 8C shows that "single-cell RNA sequencing" has the highest frequency of co-occurrence, followed by "rheumatoid arthritis" and "spinal cord injury". Among the top 20 keywords, "single-cell RNA sequencing", "rheumatoid arthritis", and "machine learning" have the highest centrality scores. These nodes act as crucial bridges or hubs in the network.

Analyzing emerging trends and focal points within the research domain of "scRNA-seq in MSK diseases" is imperative for directing funding and fostering collaborations that resonate with contemporary research imperatives. Figure 8D illustrates a timeline that maps keyword frequency clustering in critical research sectors. This graphical depiction employs circles of varying sizes to denote the annual frequency of keyword utilization, interconnected by lines that signify co-occurrences. The color scheme serves a functional purpose: purple denotes early-emerging keywords, yellow signifies recent ones, while mixed hues indicate keywords that have maintained relevance over time. Rose-colored nodes accentuate keywords of paramount importance within the network. Keywords within each cluster are sequentially arranged in chronological order from left to right. This visual format aids in elucidating the distribution and prominence of keywords across their respective clusters, with the circle's size reflecting its relative significance. It further offers a visual synopsis of the temporal range of keyword occurrences across each cluster. Keywords are organized into 12 distinct clusters for analytical clarity, including: #0 rheumatoid arthritis, #1 spinal cord injury, #2 synovial fibroblasts, #3 mesenchymal stem cells, #4 intervertebral disc, #5 machine learning, #6 T cells, #7 intervertebral disc degeneration, #8 mass cytometry, #9 tumor microenvironment, #10 bone tumors, #11 immune infiltration. These trends are vital for both researchers and industry professionals, as they reveal potential directions for future research and investment opportunities.

The results of the keyword analysis in "scRNA-seq in MSK diseases" research underscore the evolving priorities within the scientific community. They provide a vital resource for directing research activities, shaping policy, and strategizing in the industry, ensuring that initiatives align with the latest and most significant trends in the field.

### Related Diseases

**Figure 9:**
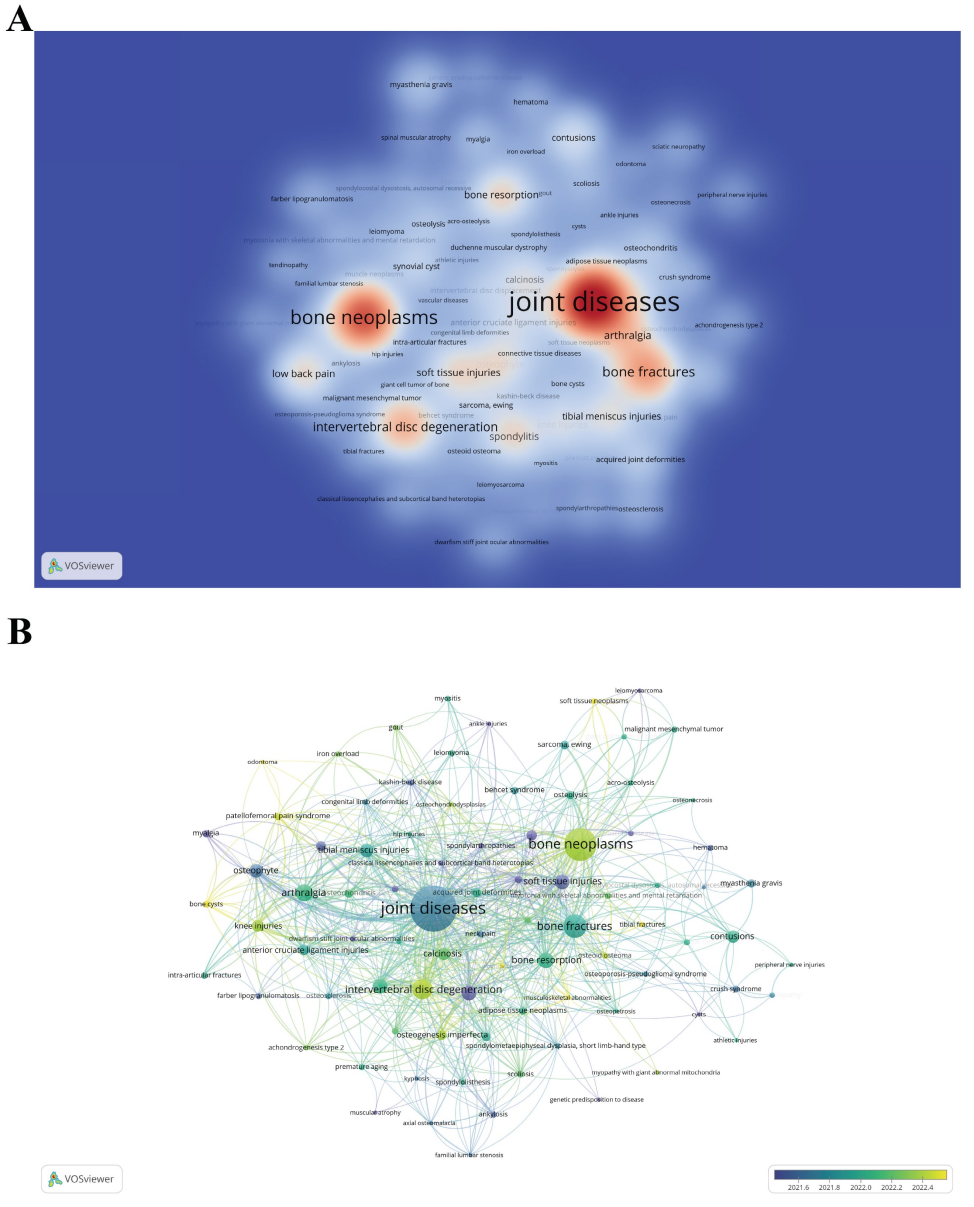
(A) Density visualization map depicting associated diseases, with color intensity reflecting the frequency of each disease. (B) Time atlas of disease occurrence. Each node, consisting of a circle and a label, varies in size according to the frequency of the disease's occurrence. The color of each sphere, as explained by the color gradient in the lower right corner, denotes the average year of occurrence; blue signifies earlier emergence, while yellow denotes later emergence.

By identifying the diseases most related to "scRNA-seq in MSK diseases" research, scientists can focus on specific health issues, potentially accelerating the development of targeted pharmaceuticals and therapies. The Citexs Data Platform has listed 687 distinct diseases mentioned in 619 academic articles, with each disease cited in at least three publications. A heatmap generated by VOSviewer visualized these diseases, showing their frequency and interconnections in "scRNA-seq in MSK diseases" research (Figure 9A). The four most frequent diseases are joint diseases, bone neoplasms, bone fractures, and intervertebral disc degeneration. Additionally, VOSviewer conducted a time atlas analysis of these diseases based on co-occurrence (Figure 9B). In this graphical representation, each node is represented by a circle accompanied by a corresponding label. The diameter of each circle correlates with the frequency at which the disease is referenced in the scholarly literature, thus offering a quantitative measure of its prevalence in academic discussions. Additionally, the hue of each circle denotes the mean year of citation, with a color gradient legend positioned in the lower right-hand section of the graphic. Blue indicates earlier disease emergence, while yellow indicates later emergence. The figure shows that among the top 20 diseases, "spondylitis" and "congenital neuromuscular disease with uniform type 1 fiber" emerged earlier (2021-2022); "intervertebral disc degeneration", "osteogenesis imperfecta", and "bone neoplasms" occurred more frequently between 2022 and 2023, indicating the recent research focus.

Understanding these trends sharpens research focus and aligns therapy and policy development with key needs in "scRNA-seq in MSK diseases" research. This alignment enhances the potential for significant discoveries and effective treatment development. Policymakers can use this data to prioritize healthcare policies and funding for frequently studied diseases like intervertebral disc degeneration and bone neoplasms. Concentrating resources on these high-impact areas could advance treatments and potentially lessen the healthcare burden of these conditions. Researchers receive data highlighting diseases common in "scRNA-seq in MSK diseases" studies, guiding their focus towards conditions such as osteogenesis imperfecta and bone fractures. This focused approach boosts research efficiency and enhances the relevance and applicability of findings. Industry practitioners can align their development strategies with diseases identified as current research hotspots, especially in pharmaceuticals and medical devices. Insights into disease trends can drive innovation in treatment solutions, ensuring new products meet pressing clinical needs.

### Challenges and Future Vistas

#### Challenges Facing "scRNA-seq in MSK Diseases"

In the evolving field of MSK research, scRNA-seq has ushered in a new era, enabling precise transcriptomic exploration at the single-cell level with unparalleled accuracy. This powerful tool has significantly enhanced our understanding of the fundamental mechanisms underlying various diseases. However, scRNA-seq faces several obstacles in MSK research that need to be overcome. Firstly, the high cost, often exceeding $1,000 per sample including cell capture and library preparation^10^, poses a challenge for widespread adoption in low- and middle-income countries with limited healthcare funding.

Secondly, the initial step in scRNA-seq is the extraction of single cells. Obtaining sufficient and high-quality RNA from stroma-rich bone tissue poses significant challenges, especially in small animals^51^. Sometimes, extracting enough viable cells from bone marrow or bone tissue is crucial as these cells accurately represent the body's cellular diversity. For instance, multiple displacement amplification (MDA) is the preferred method for amplifying genomic DNA in clinical samples with low DNA quantities^52^. However, MDA has issues such as amplification bias and uneven genome coverage^53^. Future research should focus on optimizing sample preparation techniques to facilitate the efficient extraction of cells from complex tissue matrices while ensuring the preservation of cellular viability and structural integrity. Moreover, it is necessary to improve bioinformatics algorithms, and the development of new approaches is essential for the standardized identification of novel cell types^54^.

Thirdly, the storage, sharing, and computational demands of scRNA-seq datasets have become major bottlenecks due to their large size and complexity. Advanced bioinformatics analyses can identify critical elements in the data surge. For instance, Cell BLAST, developed by Cao *et al.*^55^, enables precise and rapid retrieval and annotation of new single-cell data within existing databases. This significantly improves the accuracy and efficiency of the process, facilitating the analysis of scRNA-seq data. It is crucial to address the challenges of processing high-dimensional data. Artificial intelligence (AI), especially machine learning and deep learning, will play an important role in processing and interpreting scRNA-seq data^56^, ^57^. AI-driven approaches will automatically identify cell types, predict cell states, and discover new cellular subpopulations. Automated data annotation and integration from various datasets (e.g., spatial transcriptomics, proteomics) will simplify biological discoveries and help clarify functional relationships in complex biological systems^58^. These advancements will empower researchers to more efficiently derive biologically significant insights from complex datasets. However, rigorous biological validation is essential to confirm the authenticity of these findings and assess their clinical relevance.

Fourthly, the variability in outcomes of MSK disease studies often results from different sampling protocols, sequencing techniques, and analytical strategies used in scRNA-seq studies^24^. Additionally, poor data-sharing practices significantly reduce the value of research resources. The prevalent issue of data inconsistency often stems from the absence of standardized operating procedures across diverse laboratories. To rectify this, forthcoming initiatives should concentrate on establishing explicit standards and specifications that guarantee data uniformity across multiple research environments. The formulation of globally accepted standardization protocols and the advocacy for common data sharing principles are imperative for the progression of this scientific field. AI-based data compression techniques are crucial for reducing the size of scRNA-seq datasets without sacrificing crucial biological information. For example, neural network-based autoencoders and attention mechanisms are used to compress dataset sizes while retaining features essential for tasks like cell clustering and classification, as shown by scCompressSA and scCross^59^, ^60^. Compression algorithms using generative AI methods will enable researchers to handle large datasets more effectively and improve sharing and collaboration on large-scale scRNA-seq projects. Advanced neural networks and cloud-based AI platforms will make access to advanced computational resources more accessible, allowing smaller labs and institutions to use AI for comprehensive scRNA-seq studies. Advocating for multi-center collaborations emerges as a crucial strategy to bolster the reliability of scRNA-seq applications within MSK research. By promoting uniform adherence to standardized methodologies, researchers can secure the reproducibility of their results and establish a robust infrastructure for data sharing.

Fifth, despite its powerful applications in research, scRNA-seq is rarely used in clinical settings due to its time-consuming and complex analysis. The subsequent step involves leveraging scRNA-seq data to develop personalized treatment strategies for MSK diseases. This process will likely entail identifying crucial cellular populations or signaling pathways unique to individuals, which will guide tailored therapeutic approaches for conditions like OA, RA, and bone tumor management. AI will play a critical role in translating scRNA-seq findings into precision medicine applications^61^. By identifying individual patient profiles and predicting therapy responses, AI will facilitate the customization of treatments at the cellular level.

Sixth, scRNA-seq is advancing our understanding of cellular heterogeneity in bone, cartilage, and adjacent tissues. However, single-modality analysis often limits insights into the full spectrum of molecular mechanisms. Combining scRNA-seq with other omics technologies such as proteomics, epigenomics, and metabolomics provides a more holistic view of cellular processes in MSK research. For example, integrating RNA sequencing with proteomic analysis helps elucidate the relationship between gene expression and protein function in bone and joint diseases. AI will play a vital role in integrating scRNA-seq with other omics data (e.g., epigenomics, proteomics, and metabolomics) to offer a more comprehensive understanding of cellular function^58^. AI will enable the integration of data from multiple modalities, revealing interactions between gene expression, protein regulation, and metabolic activity, leading to a deeper understanding of cellular biology.

Seventh, while scRNA-seq provides snapshots of cellular states, it often fails to capture dynamic processes or predict future cellular behaviors. Consequently, AI models are being developed to predict cellular trajectories, responses to stimuli, and differentiation pathways^62^. These predictive models will be invaluable in simulating disease progression, tissue regeneration, and drug responses. AI-based temporal modeling of single-cell data will allow researchers to more effectively understand dynamic cellular processes and predict cell state transitions, essential for disease modeling and therapeutic interventions.

#### Future Outlook I: AI and scRNA-seq in MSK Diseases

From the previous analysis, it becomes evident that AI is poised to play a pivotal role in advancing the field of "scRNA-seq in MSK diseases" in multiple facets. It emerges as a likely key driving force for the future development of this area, particularly in the domain of data analysis^63^. Therefore, it is essential to further underscore the significance of AI in the analysis of scRNA-seq data.

1. Automation of Data Preprocessing and Quality Control: AI tools streamline preprocessing procedures, saving substantial time and resources: (1) Noise Reduction: Machine learning algorithms efficiently identify and eliminate technical noise, thereby improving data integrity^64^, ^65^. (2) Correction of Batch Effects: AI-driven methods detect and rectify batch effects, ensuring uniformity across datasets generated under varying experimental conditions or from different platforms. (3) Outlier Identification: Anomaly detection models automatically flag erroneous cells or genes, enabling researchers to focus on biologically meaningful data.

2. Enhanced Cell-Type Annotation and Classification: AI accelerates the identification of cell types and states: (1) Supervised Learning: Models trained on annotated datasets provide precise cell type classifications, avoiding the labor-intensive and time-consuming manual labeling process. (2) Unsupervised Clustering: AI-based clustering techniques, such as deep learning and variational autoencoders, reveal previously unidentified cell subtypes and states within complex tissues^66^. (3) Transfer Learning: Pretrained models enable efficient cross-dataset annotation, significantly reducing repetitive efforts across studies.

3. Improving Scalability for Large Datasets: AI facilitates the management of large-scale scRNA-seq datasets: (1) Dimensionality Reduction: AI-enhanced versions of techniques such as t-SNE, UMAP, and others reduce the complexity of high-dimensional data while retaining biological variance^67^-^69^. (2) Distributed Computing: AI frameworks optimized for parallel processing enable the scaling of analyses, significantly shortening computational times.

4. Advanced Trajectory and Lineage Reconstruction: AI aids in the study of cellular dynamics: (1) Time-Series Modeling: Recurrent neural networks and other temporal models reconstruct transitions in cell states, providing insights into differentiation pathways^70^. (2) Probabilistic Inference: Bayesian approaches leverage AI to estimate lineage hierarchies with high certainty, contributing to the understanding of developmental and disease processes^71^.

5. Reducing Manual Effort and Expertise Barriers: AI reduces the reliance on specialized computational expertise: (1) User-Friendly Interfaces: AI-driven platforms with intuitive graphical interfaces make scRNA-seq analysis accessible to a broader range of researchers. (2) Automated Pipelines: Predefined pipelines streamline the process from data preprocessing to result visualization, enhancing overall productivity.

6. Fostering Interdisciplinary Collaboration: AI facilitates collaboration between computer science, biology, and related disciplines^63^, ^72^. (1) Mutual Expertise: The collaboration between AI specialists and biomedical researchers ensures computational methods address real biological questions^73^. (2) Co-Creation: Collaborative projects stimulate innovation, resulting in the development of AI tools tailored specifically for scRNA-seq applications. (3) Knowledge Transfer: Training programs support cross-disciplinary education, enabling biologists to acquire computational skills and AI researchers to understand biomedical challenges^74^, ^75^. (4) Clinical Translation: Collaboration between AI developers and clinicians accelerates the practical application of scRNA-seq discoveries to clinical diagnostics, treatment strategies, and precision medicine^76^.

#### Future Outlook II: scRNA-seq and Precision Medicine in MSK Diseases

ScRNA-seq represents an advanced large-scale, high-throughput technique that enables comprehensive transcriptome analysis at single-cell resolution. It has become an indispensable tool in life sciences, unraveling complex cellular interactions and providing critical insights that will guide the development of personalized and targeted therapeutic approaches^77^. The transformative potential of this technology is particularly evident in the following areas:

1. Advancing Understanding of Pathogenesis: ScRNA-seq offers unprecedented detail in profiling transcriptomes at the single-cell level, making it instrumental in exploring the cellular heterogeneity of MSK tissues^78^. For disorders like OA, OP, or tendon injuries, the technique facilitates the identification of disease-specific cellular subtypes and their contributions to pathogenesis^29^, ^30^, ^33^. Notably, the discovery of previously uncharacterized stromal and immune cell subsets, along with the analysis of their molecular communication networks, has the potential to uncover novel therapeutic targets.

2. Biomarker Discovery: Precision medicine relies on biomarkers for diagnosis, prognosis, and treatment monitoring. ScRNA-seq enables the identification of biomarkers specific to cell types and contexts. For example, differential gene expression in chondrocytes, osteoblasts, or macrophages can provide predictive insights into disease progression and therapeutic responses^10^, ^24^. Furthermore, linking these biomarkers to distinct cellular states or developmental trajectories enhances their applicability in clinical settings, paving the way for more precise interventions.

3. Development of Personalized Therapeutics: The application of scRNA-seq in MSK conditions allows for the customization of treatment plans by predicting individual responses to existing therapies. For instance, single-cell analyses can evaluate the efficacy of anti-inflammatory drugs by profiling immune cell dynamics during treatment^79^, ^80^. Additionally, findings from scRNA-seq can facilitate the creation of advanced biologics, such as monoclonal antibodies or RNA-based therapeutics, which precisely target disease-driving pathways or cellular subsets^81^.

4. Applications in Regenerative Medicine: In regenerative medicine for MSK disorders, scRNA-seq can refine stem cell-based therapies by monitoring the differentiation and integration of transplanted cells at a single-cell resolution^82^. This enables the optimization of protocols for the repair of cartilage, bone, and tendon tissues^83^. Moreover, the insights provided by scRNA-seq can inform the design of bioengineered scaffolds by identifying key molecular signals that enhance tissue repair and vascularization^84^.

5. Overcoming Drug Resistance: Drug resistance, a significant challenge in conditions such as RA and bone metastases, can be better understood through scRNA-seq^77^, ^85^, ^86^. This technology identifies resistant cell populations and elucidates their adaptive mechanisms, such as metabolic alterations or changes in gene expression. Targeting these resistant subsets with tailored therapies holds the promise of significantly improving treatment outcomes.

In summary, scRNA-seq stands at the forefront of innovation in precision medicine for MSK disorders. By elucidating disease mechanisms, enabling biomarker discovery, and advancing therapeutic strategies, it offers the potential to transform clinical outcomes. As interdisciplinary collaboration and technological progress continue, scRNA-seq is poised to become a cornerstone in the paradigm of MSK precision medicine.

### Strengths & Limitations

Diverging from prior research that predominantly utilized meta-analyses or narrative reviews, this study's application of scientometric tools has provided a more comprehensive and nuanced understanding of research trends and focal areas^87^. This research is one of the first in recent years to employ a scientometric analysis to delineate the knowledge landscape of "scRNA-seq in MSK diseases". While acknowledging its inherent limitations, this study provides a broad and objective benchmark for guiding future research progress.

This study faced several limitations. First, there was a selection bias due to the constraints of CiteSpace, which limited data collection to publications indexed in the WoSCC^88^. Second, relying on citation counts as a metric for a paper's influence can lead to potential inaccuracies because of various confounding variables^89^. Third, the large number of papers reviewed might have prevented a comprehensive analysis of each document and its subfields, possibly affecting the study's thoroughness. Fourth, the employment of natural language processing within scientometric methodologies, as evidenced in prior research, is susceptible to introducing biases^90^-^92^. Fifth, although focusing on English-language texts may cause publication bias^46^, ^93^, this doesn't significantly impact the global applicability of our findings, considering the dominance of English in scientific research. Future research should incorporate multilingual databases to obtain more inclusive insights. It is suggested that subsequent studies use additional databases, such as the China National Knowledge Infrastructure and SinoMed, to explore regional or language-specific trends compared to English-language research. Ultimately, an incomplete aggregation of literature could result in the inadvertent omission of recent publications and pivotal terms throughout the data analysis phase.

## Conclusion

The burgeoning interest in scRNA-seq applications for MSK diseases highlights the urgent need to review its historical development, assess significant achievements, and map out pathways for future exploration. Despite this demand, no comprehensive scientometric analysis has been carried out to date. Filling this gap, our study pioneers a systematic and visually enriched examination of the developmental trajectory and current frontiers of scRNA-seq research in MSK diseases. Through an innovative approach, we explore geographical, institutional, and authorial dimensions, providing a detailed understanding of the advances and collaborative dynamics shaping this field. By analyzing journal distributions and co-citation networks, we reveal the research's influence, potential, and primary orientations. Additionally, our study identifies emerging research trends and projects future trajectories through a meticulous examination of co-cited literature, high-impact publications, and evolving keyword patterns. With advancements in scRNA-seq technology, we anticipate broader applications and enhanced analytical precision, paving the way for transformative diagnostic and therapeutic strategies in MSK diseases. Our findings not only present a systematic and comprehensive overview of the scRNA-seq landscape but also serve as a valuable resource for researchers, offering practical insights to guide future efforts. By clarifying the field's current dynamics and highlighting promising avenues, our work promotes a deeper understanding of pathogenic mechanisms, supports the development of precision medicine, and addresses crucial challenges in MSK research. Ultimately, these contributions are expected to significantly advance the prevention and treatment of MSK diseases, driving meaningful clinical and translational breakthroughs.

## Supplementary Material

Supplementary table.

## Abbreviations

AI: artificial intelligence
MDA: multiple displacement amplification
MeSH: Medical Subject Headings
MSK: musculoskeletal
OA: osteoarthritis
OP: osteoporosis
RA: rheumatoid arthritis
RQs: research questions
scRNA-seq: single-cell RNA sequencing
WoSCC: Web of Science Core Collection
