# Supplementary material for: Research Trends and Dynamics in Single-cell RNA Sequencing for Musculoskeletal Diseases: A Scientometric and Visualization Study
Source: Int J Med Sci. 2025 Jan 1;22(3):528–50. doi: 10.7150/ijms.104697 (PMC11783068; doi:10.7150/ijms.104697)
Supplement: Supplementary file 1 — Supplementary table. [file ijmsv22p0528s1.pdf]

Supplementary Material

1. Search strategy

Search	Query
#1	TS=(“single-cell RNA sequencing” OR “single cell RNA sequencing” OR “single-cell RNA-seq” OR “scRNA-seq” OR “single-cell sequencing” OR “single-cell transcriptomic” OR “single-cell ATAC” OR “single-cell omics sequencing” OR “single-cell RNA-sequencing” OR “Single-cell transcriptome” OR “single-cell transcriptomics” OR “single-cell multiomics sequencing”)
#2	TS=(“Osteoarthritis” OR “Osteoarthritides” OR “Osteoarthrosis” OR “Osteoarthroses” OR “Arthritis Degenerative” OR “Arthritides Degenerative” OR “Degenerative Arthritides” OR “Degenerative Arthritis” OR “Degenerative Arthritis” OR “Osteoarthrosis Deformans” OR “Osteoporosis” OR “Osteoporoses” OR “Osteoporosis Post-Traumatic” OR “Osteoporosis Post Traumatic” OR “Post-Traumatic Osteoporoses” OR “Post-Traumatic Osteoporosis” OR “Osteoporosis Senile” OR “Osteoporoses Senile” OR “Senile Osteoporoses” OR “Senile Osteoporosis” OR

	"Osteoporosis Age-Related" OR "Osteoporoses Age-Related" OR "Age-Related Osteoporosis" OR "Age-Related Osteoporoses" OR "Osteolysis" OR "Osteolyses" OR "bone infection" OR "bone infections" OR "Arthritis Rheumatoid" OR "Rheumatoid Arthritis" OR "osteosarcoma" OR "Osteosarcomas" OR "Osteosarcoma Tumor" OR "Osteosarcoma Tumors" OR "Tumor Osteosarcoma" OR "Tumors Osteosarcoma" OR "Sarcoma Osteogenic" OR "Osteogenic Sarcomas" OR "Sarcomas Osteogenic" OR "Osteogenic Sarcoma" OR "Spinal Cord Injuries" OR "Spinal Cord Trauma" OR "Cord Trauma, Spinal" OR "Cord Traumas, Spinal" OR "Spinal Cord Traumas" OR "Trauma, Spinal Cord" OR "Traumas, Spinal Cord" OR "Myelopathy, Traumatic" OR "Myelopathies, Traumatic" OR "Traumatic Myelopathies" OR "Traumatic Myelopathy" OR "Injuries, Spinal Cord" OR "Cord Injuries, Spinal" OR "Cord Injury, Spinal" OR "Injury, Spinal Cord" OR "Spinal Cord Injury" OR "Spinal Cord Transection" OR "Cord Transection, Spinal" OR "Cord Transections, Spinal" OR "Spinal Cord Transections" OR "Transection, Spinal Cord" OR "Transections, Spinal Cord" OR "Spinal Cord Laceration" OR "Cord Laceration, Spinal" OR "Cord Lacerations, Spinal" OR "Laceration, Spinal Cord" OR "Lacerations, Spinal Cord" OR

	“Spinal Cord Lacerations” OR “Post-Traumatic Myelopathy” OR “Myelopathies, Post-Traumatic” OR “Myelopathy, Post-Traumatic” OR “Post Traumatic Myelopathy” OR “Post-Traumatic Myelopathies” OR “Spinal Cord Contusion” OR “Contusion, Spinal Cord” OR “Contusions, Spinal Cord” OR “Cord Contusion, Spinal” OR “Cord Contusions, Spinal” OR “Spinal Cord Contusions” OR “cartilage injuries” OR “cartilage injury” OR “bone defects” OR “bone defect” OR “Fractures, Bone” OR “Bone Fracture” OR “Fracture, Bone” OR “Broken Bones” OR “Bone, Broken” OR “Bones, Broken” OR “Broken Bone” OR “Bone Fractures” OR “Spiral Fractures” OR “Fracture, Spiral” OR “Fractures, Spiral” OR “Spiral Fracture” OR “Torsion Fractures” OR “Fracture, Torsion” OR “Fractures, Torsion” OR “Torsion Fracture” OR “Intervertebral Disc Degeneration” OR “Degeneration, Intervertebral Disc” OR “Disc Degeneration, Intervertebral” OR “Intervertebral Disc Degenerations” OR “Disc Degeneration” OR “Degeneration, Disc” OR “Disc Degenerations” OR “Intervertebral Disk Degeneration” OR “Degeneration, Intervertebral Disk” OR “Disk Degeneration, Intervertebral” OR “Intervertebral Disk Degenerations” OR “Disk Degeneration” OR “Degeneration, Disk” OR “Disk Degenerations” OR “Disk Degradation” OR “Degradation, Disk” OR “Disk

	Degradations" OR "Degenerative Disc Disease" OR "Degenerative Disc Diseases" OR "Disc Disease, Degenerative" OR "Disc Degradation" OR "Degradation, Disc" OR "Disc Degradations" OR "Degenerative Intervertebral Discs" OR "Degenerative Intervertebral Disc" OR "Disc, Degenerative Intervertebral" OR "Intervertebral Disc, Degenerative" OR "Degenerative Intervertebral Disks" OR "Degenerative Intervertebral Disk" OR "Disk, Degenerative Intervertebral" OR "Intervertebral Disk, Degenerative" OR "Arthritis, Gouty" OR "Gouty Arthritis" OR "Arthritides, Gouty" OR "Gouty Arthritides" OR "Spondylitis, Ankylosing" OR "Bechterew's Disease" OR "Bechterews Disease" OR "Marie-Struempell Disease" OR "Marie Struempell Disease" OR "Spondylarthritis Ankylopoietica" OR "Spondyloarthritis Ankylopoietica" OR "Ankylosing Spondylitis" OR "Ankylosing Spondylarthritis" OR "Ankylosing Spondylarthritides" OR "Spondylarthritides, Ankylosing" OR "Spondylarthritis, Ankylosing" OR "Ankylosing Spondyloarthritis" OR "Ankylosing Spondyloarthritides" OR "Spondyloarthritides, Ankylosing" OR "Spondyloarthritis, Ankylosing" OR "Spondylitis Ankylopoietica" OR "Bechterew Disease" OR "Rheumatoid Spondylitis" OR "Spondylitis, Rheumatoid" OR Sarcopenia OR Sarcopenias OR

	Rhabdomyolysis OR Rhabdomyolyses OR "musculoskeletal diseases" OR "musculoskeletal disease" OR "Orthopedic Disorders" OR "Orthopedic Disorder" OR "Orthopaedic Disorders" OR "Orthopaedic Disorder" OR "Orthopedic Diseases" OR "Orthopedic Disease" OR "Orthopaedic Diseases" OR "Orthopaedic Disease" OR "musculoskeletal Disorders" OR "musculoskeletal Disorder")
#3	#1 AND #2
